# MicroRNAs: circulating biomarkers for the early detection of imperceptible cancers via biosensor and machine-learning advances

**DOI:** 10.1038/s41388-024-03076-3

**Published:** 2024-06-05

**Authors:** Gavin A. D. Metcalf

**Affiliations:** School of Life Sciences, Faculty of Science and Engineering, Anglia Ruskin University, Cambridge, UK

**Keywords:** Cancer screening, Cancer

## Abstract

This review explores the topic of microRNAs (miRNAs) for improved early detection of imperceptible cancers, with potential to advance precision medicine and improve patient outcomes. Historical research exploring miRNA’s role in cancer detection collectively revealed initial hurdles in identifying specific miRNA signatures for early-stage and difficult-to-detect cancers. Early studies faced challenges in establishing robust biomarker panels and overcoming the heterogeneity of cancer types. Despite this, recent developments have supported the potential of miRNAs as sensitive and specific biomarkers for early cancer detection as well as having demonstrated remarkable potential as diagnostic tools for imperceptible cancers, such as those with elusive symptoms or challenging diagnostic criteria. This review discusses the advent of high-throughput technologies that have enabled comprehensive detection and profiling of unique miRNA signatures associated with early-stage cancers. Furthermore, advancements in bioinformatics and machine-learning techniques are considered, exploring the integration of multi-omics data which have potential to enhance both the accuracy and reliability of miRNA-based cancer detection assays. Finally, perspectives on the continuing development on technologies as well as discussion around challenges that remain, such as the need for standardised protocols and addressing the complex interplay of miRNAs in cancer biology are conferred.

## Challenges in early detection of difficult-to-detect cancers

Current trajectories suggest that global cancer cases will reach over 35 million by 2050 [1] and that almost half of all cancer cases that were diagnosed in England in 2018 were at advanced stages (3 and 4) [2]. This highlights the urgency of early detection in order to attempt to reduce the burden this will inevitably cause on patients, their social support network, healthcare systems, as well as local and global economies.

However, despite this pressing need, early detection poses many challenges. The lack of systemic effects due to the small size of primary tumours, or metastases, in early stages of development often leads to the asymptomatic nature of disease progression. In addition, the reported lack of specific and sensitive screening tests that follow standardised testing parameters collectively contribute to delayed accurate detection.

Certain well-documented cancers present unique challenges in diagnosis, including pancreatic, non-small cell lung cancer (NSCLC), liver, and central nervous system (CNS) tumours. All aforementioned cancers share the challenge of being asymptomatic in early stages of development, and often present with vague and non-specific symptoms as the disease advances [3–7]. The deep-seated retroperitoneal location of the pancreas frequently hinders early detection of cancer through common screening methods. In addition to this, there are no widely adopted reliable screening tests [8]. NSCLC has limited effective screening tools applicable to high-risk groups, high false positive rates in common imaging techniques along with invasive diagnostic biopsy procedures posing significant risks, especially in light of the recent pandemic [9, 10]. The early detection of Liver cancers is complicated by cirrhosis masking key diagnostic symptoms, imaging limitations when considering small lesions due to poor arterial phase hyperenhancement of contrast agents and the challenges of liver biopsies [11, 12]. CNS tumours often present with subtle or non-specific symptoms due to their location, imaging struggles to differentiate between benign and malignant tumours and biopsy procedures carry high risks [13, 14].

These reasons, combined with technological advances in biosensor and machine-learning development, have led to heightened interest in the area of clinically pertinent microRNA (miRNA) biomarkers obtained from, and stable within, minimally invasive biofluids, such as blood, saliva, and urine.

## MicroRNAs

MicroRNAs (miRNAs) are short, non-coding RNA molecules that are typically 18–25 nucleotides in length. The first miRNA, *Lin-4*, was discovered in *Caenorhabditis elegans (C. elegans)* in 1993 by Ambros and colleagues. Initial results suggested that the *Lin-4* gene did not code for a protein but, instead, gave rise to a short RNA sequence of 22 nucleotides in length that was observed to interact with the 3′ untranslated region (UTR) of the Lin-4 messenger RNA (mRNA), via Watson-Crick base pairing, repressing its expression and initiating the larval stages of development [15]. Research into miRNAs continued at a somewhat slow pace and in 2000 another pivotal miRNA was reported by Reinhart and colleagues. Let-7 was identified as a key player in the timing of larval development in the same model system, *C. elegans*. Notably, Let-7 is evolutionarily conserved across diverse species, including *Homo Sapiens*, indicating a fundamental role in biological processes [16]. The discoveries of Lin-4 and Let-7 collectively reshaped the scientific landscape, leading to a new field of research with substantial potential to impact development, health, and disease across a wide range of species. Upon consideration of cancer, a pioneering study by Calin and colleagues shed light on the aberrant expression of specific miRNAs in Chronic Lymphocytic Leukaemia (CLL). In particular, *miR-15a* and *miR-16-1* genes, often down-regulated or deleted in approximately 68% of CLL cases, were found to target the *B-cell lymphoma 2* (*BCL-2*) gene, providing a mechanistic link between their downregulation or deletion and the apoptotic resistance commonly observed in CLL cells. Thus, suggesting that the loss of these miRNAs contributes to the pathogenicity of CLL, via promotion of cell survival [17]. The findings resulted in a growing field of research, contributing to a broader understanding of the roles of miRNAs in cancer biology, offering potential avenues for the development of miRNA-based therapeutics, as well as pioneering efforts to identify miRNA signatures that could be used to distinguish between different cancer types.

MiRNAs are coded for in both exons and introns of genes or non-coding RNA transcripts [18] suggesting a link to host-gene promoters. MiRNA-coding sequences are first transcribed by RNA polymerase II (Pol II) into long primary transcripts (pri-mRNAs) [19]. Pri-mRNAs are then capped and polyadenylated [20], and subsequently cleaved in the nucleus by Drosha and Pasha resulting in a hairpin-looped precursor nucleotide (pre-miRNA) of approximately 60–75 nucleotides in length. Pre-miRNA is actively transported out of the nucleus into the cytoplasm by Exportin 5 [21] whereupon another RNAse III enzyme, Dicer, binds and cleaves the pre-miRNA generating a 19 to 23 nucleotide duplex structure with a mature (master) and complementary (passenger) strand. The mature miRNA is then integrated into the RNA-induced silencing complex (RISC) chiefly consisting of Argonaute Proteins 1–4 (AGO 1–4), Dicer, and TAR RNA-binding protein (TRBP) [22], which guides the RISC to specific mRNA targets, leading to either translational repression or mRNA degradation [23].

### Circulating microRNAs

Freely circulating nucleic acids in blood were first denoted about 60 years ago [24, 25], with subsequent research reporting that tumour-specific DNA and RNA were frequently found in the plasma of cancer patients [26, 27]. Historically it was a common belief within the scientific community that RNA molecules would not be a suitable biomarker within blood samples due to the presence of endogenous nucleases within plasma [28], however upon the discovery of miRNAs within fixed tissues [29] this idea was rapidly dismissed. This was further explored by Chen et al. [30] who identified a set of miRNAs that were consistently present and stable within serum samples. This groundbreaking work laid the foundation for the exploration of circulating miRNAs as non-invasive biomarkers for various diseases, including cancer.

**Fig. 1:**
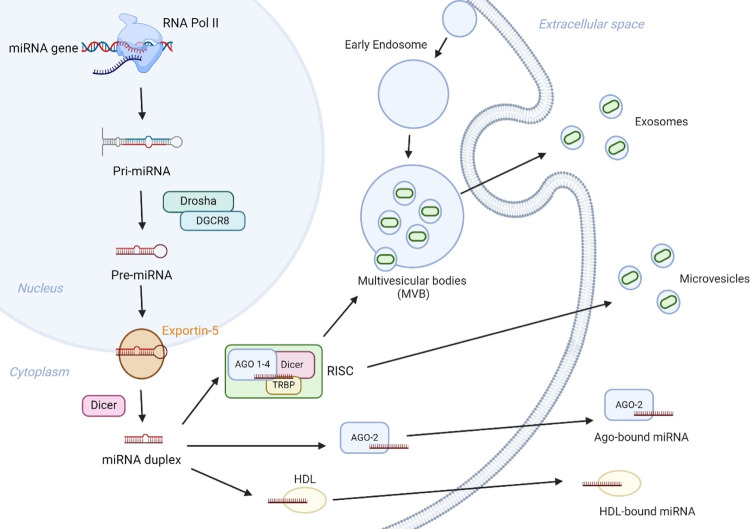
MiRNA excretion mechanisms and extracellular survival. MiRNAs are actively and passively released by cells via various approaches, including multivesicular bodies (MVB) and cellular excretion via exosomes, microvesicle formation achieved via membrane shedding, as well as association with AGO-2 and HDL.

The release of miRNAs from cells into extracellular environments, including blood, is believed to result from a variety of mechanisms. These include active secretion via exosomes, microvesicle release, and protein-mediated export (such as high-density lipoproteins and AGO2) [31–34] (Fig. 1). The stability of circulating miRNAs is enhanced by their association with various carriers, as described, protecting them from nuclease- and protease-facilitated degradation [35, 36] as well as stabilising the molecules when exposed to sample processing conditions such as freeze-thaw cycles [37].

The first reported case of using miRNAs as candidate biomarkers for cancer was published by Lawrie and colleagues in 2008 whereby blood serum concentrations of a panel of three miRNAs (miR-155, miR-210 and miR-21) were compared in patients with diffuse large B-cell lymphoma compared to healthy control sera [38]. This pioneering publication demonstrated for the first time that miRNAs are clearly detectable in serum samples and that miRNAs have potential for clinical approaches in cancer detection via minimally invasive means of sample sourcing.

## Recent developments of microRNAs as biomarkers for early cancer detection of difficult-to-diagnose cancers

As outlined, the emergence of miRNAs as promising biomarkers for early cancer detection has been a key development in the field of molecular biology. Further to this, particular miRNA signatures have been suggested to hold great impact when regarding efficient diagnosis of imperceptible cancers at an early stage. We shall go on to explore the identification and clinical validation of miRNA signatures, along with technological advances in biosensor development and machine learning used to detect, analyse, and report key miRNA signatures.

### Identification and clinical validation of miRNA signatures

Pioneering research by Lu et al. [39] conducted a screen of expression profiles of miRNAs from more than 300 patient-derived biosamples, including multiple cancers. They were able to successfully classify poorly differentiated tumours using miRNA profiles alone, which when compared to messenger RNA (mRNA) counterparts which were highly inaccurate, emphasised the potential of miRNA profiling in cancer diagnosis. Given the growing promise that miRNA’s serve as biosample-based diagnostic tools, the exploration of their application within the field of imperceptible cancers has been growing steadily.

In 2024, Shi et al. [40] explored miRNAs that could serve as biomarkers for the early detection of pancreatic cancer in patients presenting with chronic pancreatitis. The study successfully implemented a robust rank aggregation (RRA) machine-learning algorithm to aid in early pancreatic diagnosis via screening the expression profile of candidate miRNA biomarkers. Serum-derived miR-205-5p was identified as a promising predictor candidate that was observed to discern between patients with pancreatitis and pancreatic cancer, with reported accuracy rates of 91.5%. Furthermore, results demonstrated that miR-205-5p expression could be used as a predictive marker for more advanced disease, with high-expression rates matching with poorer prognosis within R1/2 resection margins of tumour specimens (indicative of residual tumour) compared to R0 resection counterparts (resection for cure or complete remission).

NSCLC is another well-reported cancer that is difficult to diagnose in early stages, often due to asymptomatic presentation, erroneous radiographic interpretation, or symptoms being wrongly attributed to other chronic respiratory conditions such as chronic obstructive pulmonary disease (COPD) [41]. A study by Dong et al. [42] analysed the miRNA profiles of plasma samples derived from both NSCLC patients and healthy controls via a miRNA microarray. Real time quantitative RT-q-PCR was then used to assess the expression levels of 11 different miRNAs that were upregulated. Three particular miRNAs, miR-1247-5p, miR-301b-3p and miR-105-5p, were demonstrated to accurately distinguish between patients with NSCLC and healthy individuals, with corresponding AUCs being reported as 0.769, 0.761, and 0.777, respectively. Such findings are supported by previous studies [43, 44] which denote that miR-301b-3p is not only present at elevated rates in NSCLC, but pivotal to cancer cell characteristics including proliferation, migration and invasion via targeting Rho GTPase activating protein, DLC1. Further analyses conducted by Arab et al. [45] identified elevated miR-141 expression in plasma samples from NSCLC patients and matched healthy controls, independent of age and sex. ROC plot analyses indicated high sensitivity (82.7%) and specificity (98%) for miR-141 in terms of discriminating between early NSCLC patients (TNM stages I, II and IIIA) and healthy individuals.

Liver cancer, most commonly Hepatocellular carcinoma (HCC), is commonly diagnosed at an advanced stage with poor prognosis due to limited therapeutic options. Several studies have reported the promise of miRNAs as biomarkers for early diagnosis of HCC opening up the opportunity for a greater volume of treatment options and thus heightened prognostic outcomes. Amr et al. [46] explored the promise of two previously reported miRNAs (miR-122 and miR-224) as biomarkers for early stage HCC diagnosis, in comparison to the conventional serum marker, alpha-fetoprotein (AFP), which has a modest accuracy of detecting early stage HCC [47]. Blood plasma samples of patients with HCC, preceded by chronic HCV infection (*n* = 40) were compared to non-HCC controls, those with Chronic hepatitis C (CHC) (n = 40) and disease-free individuals (*n* = 20). RT-qPCR assays specific for hsa-miR-122 and hsa-miR-224 were run to assess the expression levels within all samples obtained. Chief findings reported mean plasma values of miR-122 and miR-224 were significantly lower and higher, respectively, within HCC samples in comparison to HCC-free groups. Furthermore, results suggested that miR-122 and miR-224 could predict HCC with comparable sensitivity (87.5% and 92.5%, respectively), specificity (95 and 90%, respectively), and accuracy (0.96 and 0.94, respectively). In comparison, AFP sensitivity and specificity results were much poorer at 57.5 and 95%, respectively. Additional studies further support the findings of reduced miR-122 and elevated miR-224 expression profiles as promising biomarkers for the early diagnosis of HCC [48–50].

Despite overall cancer-derived mortality declining over the past two decades, CNS malignancies still contribute to high mortality rates [51]. This is believed to be largely due to a lack of accurate mass screening methods for early detection often hindered by factors such as the blood–brain–barrier (BBB). Cerebrospinal fluid (CSF) due to its function is in direct contact with any possible pathology within the CNS and is a model biofluid source for biomarker detection, with relatively easy sourcing via a lumbar puncture. MiRNAs have been widely reported to be abundant within CSF [52], with previous studies suggesting that dysregulated miRNA expression is associated with malignant CNS tumours [53, 54]. Promising results were reported in a small-scale study conducted by Shalaby et al. [55], whereby the promise of miRNAs were explored as novel biomarkers for medulloblastoma (MB) detection. The study explored the presence of extracellular miRNAs within both cell culture medium as well as CSF samples obtained from MB and non-tumour control patients. Microarray analysis identified 268 high-expression miRNA profiles and 6 low-expression miRNA profiles, in comparison to controls. Selected miRNAs were chosen to validate these findings (including miR-486-3p, miR-572, miR-3918, miR-4476, miR-615, miR-1290, miR-152a, miR-125b, and miR-1298) via RTq-PCR. The analysis confirmed the heightened presence of 4 key miRNAs (miR-1290, miR-125a, miR-125b and miR-1298) within CSF from test samples. This pioneering, albeit small, study identified a small selection of key miRNAs that were enriched within CSF samples from MB patients, which contributed to additional larger-scale studies being conducted. One such study was conducted by Kopkova et al. [56], which involved a two-phase (discovery and validation) approach. During the initial discovery phase 89 CSF samples, taken from patients with glioblastoma (*n* = 32), low-grade glioma (*n* = 14), meningioma (*n* = 11), brain metastases (*n* = 13) and non-tumour controls (with normal-pressure hydrocephalus, *n* = 19), were screened via small RNAseq analysis. This identified miRNAs with altered expression when compared to controls, ultimately leading to a panel of 9 candidate miRNA biomarkers (let-7a, let-7b. miR-10a, miR-10b, miR-21-3p, miR-30e, miR-140, miR-196a and miR-196b) to be explored. During the validation phase a further 126 pathology-matched CSF samples independent of the discovery cohort, including glioblastoma (*n* = 41), low-grade glioma (*n* = 8), meningioma (*n* = 44), brain metastases (*n* = 12) and non-tumour controls (with normal-pressure hydrocephalus, *n* = 21), were processed via RTq-PCR. Findings identified that combination patterns of 5 key miRNAs (miR-30e, miR-140, let-7n, miR-10a and miR-21-3p) served as promising biomarker tools in discerning between healthy donors and cancers, with high sensitivity and specificity. Furthermore, the study also identified a promising prognostic panel in glioblastoma patients, with median overall survival (OS) scores in patients with elevated levels of miR-10b and miR-196b observed to be 9 months, compared to low-expression concentrations equating to median OS of 16.5 months.

Collective outputs from key studies, inclusive of particular aforementioned studies from above, can be found in Table 1, further determining the efficacy of circulating miRNAs as biomarkers for imperceptible cancers.

**Table 1:** Circulating miRNAs as clinical biomarkers for detection of difficult-to-diagnose cancers.

miRNA profiling	Test sample	Cancer Type	Healthy Control vs. Cancer			Study Reference
			Sensitivity	Specificity	AUC (95% CI)	
miR-20a, miR-21, miR-25, miR-99a, miR-185, miR-191	Serum	Pancreatic (PDAC)	89%	100%	0.992	[85]
miR-30c-5p, miR-let7e-5p, miR340-5p, miR-223-3p, miR-26a-5p, miR-340-3p, miR-335-5p, miR-23b-3p, miR-142-3p, miR-200c-3p, miR-148a-3p, miR-216a-5p, miR-145-5p, miR-200b-3p, miR-143-3p, miR-34a-5p, miR-429, miR-141-3p, miR-1260b, miR-145-3p, miR-216b-3p, miR-200a-3p, miR-1260a, miR-217-5p	Plasma and Serum	Pancreatic (PDAC)	87%	88%	0.920	[86]
miR-93-5p, miR-339-3p, miR-425-5p, miR-425-3p	Plasma	Pancreatic (PDAC)	80%	94.7%	0.885	[87]
miR-15b, miR-27b	Serum	Lung (NSCLC)	100%	84%	0.980	[88]
miR-155, miR-20a, miR-25, miR-296, miR-126, miR-223, miR-199a, miR-24, miR-152, miR-145, miR-let7f	Plasma	Lung (NSCLC)	81.8%	82.9%	0.879	[89]
miR-31-5p, miR-210-3p, miR21-5p	Sputum and Plasma	Lung (NSCLC)	85.5%	91.7%	0.913	[90]
miR-1247-5p, miR-301b-3p, miR-105-5p	Plasma	Lung (NSCLC)	72.5%	82.2%	0.815	[42]
miR-141	Plasma	Lung (NSCLC)	96.3%	99.3%	0.972	[45]
miR-16 and miR-122	Serum	Liver (HCC)	58%	84%	0.803	[91]
miR-206, miR-141-3p, miR-433-3p, miR-1228-5p, miR-199a-5p, miR-122-5p, miR-192-5p, miR-26a-5p	Serum	Liver (HCC)	86%	73%	0.887	[92]
miR-4661-5p, miR-4746-5p	Serum	Liver (HCC)	85%	89%	0.942	[93]
miR-92-3p, miR-107, miR-3126-5p	Serum	Liver (HCC)	97.5%	87.8%	0.962	[94]
miR-30e, miR-140	CSF	CNS (Brain Tumour)	76%	75%	0.776	[56]
miR-15b	CSF	CNS (Glioma)	90%	94.9%	0.960	[95]
miR-15b	Plasma	CNS (Glioma)	100%	100%	1.000	[96]
miR-210	Serum	CNS (Glioma)	91.3%	91.27%	0.927	[97]

*miR* microRNA, *AUC* area under the curve, *PDAC* pancreatic ductal adenocarcinoma, *NSCLC* non-small cell lung cancer, *HCC* hepatocellular carcinoma; *CSF* cerebrospinal fluid, *CNS* central nervous system.

### Technological biosensor advances

Due to the nature of miRNAs being short and highly conserved, resulting in high homology, presents challenges in detection that is specific and sensitive. Overcoming such challenges relies upon technological advances in their detection, of which there have been an abundance in recent years. RTq-PCR was rapidly accepted by the scientific community as a means of quantifying miRNA, after first being described in published works by Chen et al. [57]. It is now deemed as an orthogonal and gold-standard approach for validating miRNA expression differences within sample sets. However, due to limitations of this approach, including RNA-sensitivity, specificity and cross-reactivity, and normalisation, other techniques are now becoming established as validation approaches when exploring miRNA detection and profiling. One technique, NanoString’s nCounter® microRNA assay, was reported as having advantages in terms of improved sensitivity and specificity over conventional techniques (such as RTq-PCR) within a pioneering paper in 2014 by Mestdagh et al. [58], a finding that has been further supported within the literature [59, 60]. The technology works without relying upon reverse transcription or amplification and is capable of highly multiplexed analysis of samples. This is achieved via direct digital detection of RNA or DNA molecules of interest using colour-coded pairs of probes (capture and reporter) which hybridise to the target molecules. The high-throughput approach facilitates reliable and reproducible expression profiling of up to 800 genes in a single assay, with input biomarker molecules as low as 1 ng (RNA). Another well-reported sensing technology is that of Next Generation RNA sequencing (RNAseq) as utilised in the previously discussed study conducted by Kopkova et al. [56]. RNAseq, developed upon classic Sanger sequencing, offers a parallel sequencing by synthesis (SBS) approach that has many advantages over other sequencing approaches. Not only does it offer large data outputs (300 kb up to multiple terabases/run) but has high sensitivity, quantitative precision, as well as the ability to screen a high number of samples in parallel (high throughput in nature) [61]. However, despite this there are some challenges associated when applying this technology to miRNA screening. These include misannotation of novel or underexpressed miRNA [62, 63], sequencing biases based on library preparation protocol variations, and extensive sample processing [64]. As a means of circumnavigating some of these challenges new technologies are being developed within the scientific community. Recently, Cai et al. [65] reported a molecular probe-based system that allows for amplification-free multiplexed detection of microRNAs within unprocessed biofluid samples. This electro-optical sensing platform functions via custom probes combining a DNA carrier and molecular beacon labelled with a reversible fluorophore and quencher in a hairpin structure which upon binding to the miRNA-target unhinges and restores fluorescence. The DNA-carrier acts as a barcode with its length resulting in differential electrical signal upon nanopore (nanopipette) detection. This technology displayed femtomolar sensitivity thus supporting detection of sub-nanomolar concentrations of miRNAs within biofluids without the need of amplification steps, and single-base mismatch selectivity essential for discriminating between miRNA’s high homology due to their short sequence length, on unprocessed blood serum samples obtained from Prostate Cancer patients. One limitation of this strategy is the electrical resolution of DNA with nanopore; however this could be resolved via reconstruction of nanopores using smaller pore sizes or heightened bandwidths. Furthermore, environmental conditions such as temperature and surrounding humidity could also affect the nanopipette setup. However, despite such limitations, due to the nature of this strategy, panels of known miRNA signatures could be screened simultaneously within biosamples such as serum (as reported) as well as other biosamples including CSF thus aiding towards its use for miRNA detection in imperceptible CNS cancers. Another study reported impressive limit of detection (LOD) improvements in comparison to the techniques described. Kanik et al. [66] presented a sensitive and multiplexed digital microarray setup that uses a polarisation-enhanced single-particle reflectance imaging sensor (SP-IRIS) to detect plasmonic gold nanorod probes that hybridise to miRNA targets. Within proof of concept studies, a LOD of 100 attomolar was reported when detecting synthetic miR-223-3p as a target, showing marked improvements over more common femtomolar ranges. Furthermore, the assay itself was conducted in 35 min highlighting its value within a healthcare setting for rapid diagnostic applications.

### Integration of Bioinformatics and machine learning

Bioinformatics and machine-learning approaches have shown incredible aid towards processing and interpretation of biological data. In current biomedical research wet experimentation and bioinformatics analytics are equally pivotal – with large complex datasets needing increasingly sophisticated management and analysis systems to gain biotic knowledge for therapeutic and diagnostic applications. This is true of all disease states, but especially so for cancer due to its complex and multifaceted nature. The introduction of evolutionary supervised AI learning methods is key to improving early-stage cancer diagnosis and therapeutic decisions, collectively aiding towards heightened rates of remission and overall survival. One such evolutionary learning method is Cancer*Sig* [67] which followed a bi-objective combinatorial genetic algorithm, with the end goal of identifying the miRNA signatures that could aid in early-stage detection of cancers. This was represented as: C(*n*, *m*), where ‘*m’* is a cancer stage-specific miRNA signature and ‘*n’* is a pool of 7,117 candidate miRNAs from 4,667 patients with 15 cancer types, including HCC, obtained from The Cancer Genome Atlas (TCGA). The pan-cancer analysis of miRNA signatures collectively proposed that three miRNAs (let-7i-3p, miR-362-3p and miR-3651) could discriminate between tumour and non-tumour samples and extensively contributed to stage predictions amongst 8 of the 15 cancer types analysed.

Other combined machine learning and bioinformatics approaches have been described as a means of identifying potential diagnostic pancreatic cancer-specific miRNA biomarkers [68]. The study analysed serum-derived miRNA expression profiles from three datasets (independent) obtained from the Gene Expression Omnibus (GEO) database. Collectively, three machine-learning algorithms (Support Vector Machine-Recursive Feature Elimination, Least Absolute Shrinkage and Selection Operator regression analysis, and Random Forest) identified three key candidate miRNAs (miR-4648, miR-125b-1-3p, and miR-3201) which displayed promise as diagnostic biomarkers due to exhibiting altered differential expression patterns. The combined model described exhibited notable performance and accuracy in both training and validation processes, with reported AUC values of 0.926 and 0.935, respectively.

While the promise of such approaches is putative, limitations of deep learning models within the cancer field exist. Such limitations include the lack of large phenotypically characterised open-source datasets often as a result of high processing costs and restricted sample availability. Furthermore, storage of tumour samples is typically within formalin-fixed paraffin-embedded (FFPE) blocks which commonly results in RNA degradation [69] and DNA crosslinking [70], thus making samples unsuitable for profiling and data production. AI uncertainty also needs to be considered, with most approaches resulting in point-estimate (predictive) methods, which could, when applied to the clinic, result in overconfident predictions and inaccurate diagnosis. Bayesian approaches, such as the recently reported Epistemic Invariance in Cancer Classification (EpICC) [71], could however help resolve this. Despite such limitations, the invaluable implications of (often combined) computational techniques holds great hope for accurately and promptly predicting the presence, and stage, of cancers including imperceptible neoplasm.

## Current challenges and recommendations in the use of microRNAs for early cancer detection

As described, miRNAs meet key characteristics of being deemed promising candidate clinical biomarkers due to their stable properties and ubiquity within a plethora of readily accessible biofluids, obtained via non- and minimally invasive means. Storage of biofluids is an important consideration when considering biomarkers, however, miRNAs have been reported to be considerably stable in a variety of biofluids, including blood, CSF, urine, saliva, and lacrimal fluid held at room temperature for short-term storage up to 96 h [72], and long-term storage for months at a time at low (−20 °C) and ultralow (−80 °C) temperatures [73, 74]. Furthermore, routine laboratory processes such as freeze-thaw cycles are commonly reported to not adversely affect miRNA quality [75, 76]. Conversely, technical considerations in terms of processing are frequently reported constraints when considering freely circulating miRNAs within blood samples. Studies have reported variations of miRNA concentrations in samples due to contamination by haemolysis during processing [77], as well as altered miRNA profiles as a result of platelet activation [78] – although the extent of this could be affected by donor age, gender, and race [79]. Upon consideration of the wider application of cancer biology this effect may not be particularly disruptive as known erythrocyte and platelet-derived miRNAs could be omitted from screening profiles, should a large enough pool of miRNA signatures be explored. However, when using samples for cardiovascular biomarkers, for example acute myocardial infarction where platelets play a fundamental pathophysiological role, this could diminish diagnostic value extensively [80].

While aforementioned donor age, gender and race are reported to potentially affect haemolysis rates and platelet activation cascades, physiological variables could also have an impact on miRNA concentrations, thus questioning the validity of “healthy control” samples used within studies. A number of studies have explored variables including age [81], gender [82], and BMI [83, 84] with findings suggesting altered expression profiles of particular miRNAs in comparison to counterparts. Although there is an increasing number of research outputs, there is a lack of consistent evidence of which miRNAs are associated with particular confounding factors, thereby highlighting the necessity for further research.

Circulating miRNAs concentrations are considerably lower than those within cells and tissues, presenting challenges in accurate detection and quantification. Current gold-standard approaches, including NGS, enable relatively low miRNA input values, whereas other recently developed novel approaches as discussed above have the ability to detect attomolar ranges [66]. This attempts to circumnavigate low concentrations, however due to the heterogenous nature of cancer, the variability of miRNA expression amongst populations, individuals, tissues, and cells as well as the dynamic nature of miRNA regulation, there is an absence of unanimously agreed robust internal controls. This creates a challenge that can only be truly overcome by combining several factors including agreed consensus amongst the scientific community (aligned under internationally recognised SOPs) for testing, consistent validation studies, and crucially open-access data-sharing repositories.

Lastly, while there are an ever-increasing number of techniques being reported to profile and quantify stable miRNAs obtained from biofluids, there is yet to be an optimised standard for cancer biomarker discovery within clinical settings. Furthermore, although there is a pressing need for accurate diagnostic tests, there is also a growing view within the scientific community that a turning point from the lack of sensitive and specific screening tests to increasingly sensitive detection methods can, in fact, make it difficult to distinguish between clinically insignificant changes and lesions that could result in aggressive, life-threatening, cancers. This issue, while not yet reported within the field of miRNA biomarkers, is something that needs to be carefully observed moving forward.

## Conclusion

Early detection of cancer is vital in attempts to mitigate the growing burden of the disease, with the ambition of increasing patient remission and overall survival rates. However, despite this pressing need, a myriad of challenges exist when considering imperceptible cancers including the asymptomatic nature of many tumours, comorbidities, and a lack of sensitive and specific screening tests. MiRNAs present as promising early diagnostic cancer biomarkers, with highly sensitive and specific diagnostic signatures being well reported within the literature for a variety of difficult-to-diagnose cancers such as NSCLC and HCC, as well as being able to accurately discern between patients with pancreatitis and pancreatic cancer. The field of diagnostics continues to drive forward with enhanced detection of miRNAs being reported. Commercial platforms such as Nanostring’s nCounter ® microRNA assay as well as Next Generation RNA Sequencing (RNAseq) outperform what was once considered the gold-standard method, RT-qPCR, in terms of sensitivity and specificity, with other novel sensing technologies offering even greater sensitivity and LOD rates as well as heightened multiplexing capabilities. Additionally, the integration of bioinformatics and machine learning, such as the evolutionary supervised AI learning method CancerSig, enable further identification of promising miRNA signatures via predictive modelling in a scalable and reproducible automated means. Yet, despite the promise of miRNAs as cancer biomarkers, challenges exist when considering their clinical application. Many of these challenges, although being intrinsic in nature, could be circumnavigated however, via united collaborative efforts within the scientific community, sharing datasets through open-source platforms, applying standardised protocols, and fully embracing technological advances.
